# OH-MEMA: An Integrated One Health Mixed-Effects Modeling Approach for Syndromic Surveillance

**DOI:** 10.3390/jcm15082966

**Published:** 2026-04-14

**Authors:** Aseel Basheer, Parisa Masnadi Khiabani, Wolfgang Jentner, Aaron Wendelboe, Jason R. Vogel, Katrin Gaardbo Kuhn, Michael C. Wimberly, Dean Hougen, David Ebert

**Affiliations:** 1 Data Institute for Societal Challenges (DISC), University of Oklahoma, Norman, OK 73019, USA; parisa.masnadi@ou.edu (P.M.K.); mcwimberly@ou.edu (M.C.W.); 2 Office of Responsible Artificial Intelligence (ORAI), University of Arizona, Tucson, AZ 85721, USA; wjentner@arizona.edu (W.J.); ebertd@arizona.edu (D.E.); 3 Department of Epidemiology, Fay W. Boozman College of Public Health, University of Arkansas for Medical Sciences, Little Rock, AR 72205, USA; awendelboe@uams.edu; 4 School of Civil Engineering and Environmental Science, University of Oklahoma, Norman, OK 73019, USA; jason.vogel@ou.edu; 5 Hudson College of Public Health, University of Oklahoma Health Sciences Center, Oklahoma City, OK 73117, USA; katrin-kuhn@ou.edu; 6 Department of Geography and Environmental Sustainability, University of Oklahoma, Norman, OK 73019, USA; 7 School of Computer Science, University of Oklahoma, Norman, OK 73019, USA; hougen@ou.edu

**Keywords:** one health, infectious disease epidemiology, computational modeling, collaborative surveillance, pandemic intelligence, public health data integration

## Abstract

**Background/Objectives:** Integrating heterogeneous One Health time series into transparent and usable surveillance workflows remains difficult because data preparation, modeling, and interpretation are often separated across tools. In this paper, we introduce OH-MEMA (One Health Mixed-Effects Modeling and Analytics), an interactive visual analytics framework that integrates heterogeneous One Health data streams, including human clinical outcomes, environmental factors, and wastewater surveillance data, to support syndromic surveillance and pandemic preparedness. **Methods:** The system enables users to upload and analyze multi-source datasets through an interactive web-based interface. The modeling component supports fixed effects for multi-source predictors, random effects for spatial, temporal, and demographic grouping variables, optional random slopes, and rolling time-series validation. Model results are visualized as time series comparing observed and predicted outcomes, with evaluation metrics including Mean Absolute Error (MAE), Root Mean Square Error (RMSE), and correlation. To support iterative exploration, the system incorporates analytic provenance through a visual model tree that records prior configurations. **Results:** OH-MEMA was validated through both quantitative and qualitative evaluations. Quantitatively, mixed-effects models were assessed across multiple counties and outcomes using RMSE, MAE, and correlation, demonstrating robust predictive performance. Qualitatively, expert users, including epidemiologists and disease surveillance analysts, evaluated the system using the NASA Task Load Index and open-ended interviews, indicating improved interpretability, manageable cognitive workload, and effective workflow integration. **Conclusions:** OH-MEMA provides an interpretable, human-in-the-loop platform for exploratory forecasting and comparative model analysis in syndromic surveillance. The framework effectively bridges data integration, modeling, and interpretation, supporting user-centered analytical reasoning and decision-making in One Health applications.

## 1. Introduction

Effective pandemic preparedness requires the capability to quickly detect, interpret, and respond to various health signals across human, animal, and environmental domains [1]. The One Health paradigm emphasizes the interconnectedness of multiple datasets, underscoring the need for integrated approaches to disease surveillance [2]. However, a significant challenge lies in combining diverse data sources into actionable models. This is particularly important at fine-grained spatial (e.g., county-level) and temporal (e.g., weekly) scales, where decision-makers must be able to understand and trust the information [3,4,5].

Time-series analysis is essential for identifying trends, seasonality, and anomalies in epidemic surveillance. However, the heterogeneity of multi-source datasets, such as clinical case counts, environmental indicators, and wastewater pathogen signals, poses significant challenges for harmonized temporal modeling. Variations in scale, units, and reporting cadence complicate traditional time-series methods. Moreover, when visualizing such data, combining multiple modalities can lead to visual clutter, making it difficult for analysts to interpret key signals [6]. Compared to distributed lag nonlinear models (DLNMs) [7], which explicitly parameterize exposure lag response relationships, and generalized additive mixed models (GAMMs) [8], which flexibly capture nonlinear effects through smooth functions, the proposed mixed-effects framework prioritizes interpretability and interactive model specification, enabling users to explore lag structures and heterogeneous effects within a unified and transparent analytical workflow.

Despite substantial progress in epidemic dashboards, interactive visualization tools, and predictive modeling frameworks, existing systems largely treat data integration, model construction, and interpretation as separate stages handled by different tools or expert roles [9,10]. Visualization-driven surveillance platforms primarily emphasize descriptive monitoring through charts and maps, providing situational awareness but limited analytical depth [6,11,12]. In contrast, statistical and machine-learning-based epidemic models often prioritize predictive accuracy but may present challenges for non-technical domain experts, particularly when implemented through offline workflows or requiring programming expertise [13,14]. Recent advances in explainable machine learning and interpretable modeling frameworks have improved accessibility and transparency; however, integrating these approaches into interactive, user-driven analytical workflows remains an ongoing challenge. Even existing visual analytics systems that support model comparison or ensemble forecasting rarely make modeling assumptions, temporal lag structures, or uncertainty trade-offs explicit in a way that supports iterative, human-in-the-loop reasoning [9,13]. As a result, analysts may face difficulties in systematically exploring how and why different One Health signals influence disease dynamics, which can limit trust, transparency, and the generation of actionable insights [3,4].

The COVID-19 pandemic has highlighted the potential and limitations of current epidemiological visualization approaches. For tracking and visualizing pandemic data, numerous systems were rapidly developed [11,15,16]. While several integrated frameworks have emerged, a universally adopted comprehensive analytical framework that fully integrates diverse data sources and supports real-time decision-making across all scales remains elusive. For instance, the evolution of wastewater-based disease surveillance has shown promising results as an early warning system [17,18]; however, fusing these data with clinical and demographic data remains challenging.

In this paper, we present OH-MEMA, an interactive visual analytics framework designed to assist domain experts, such as public health researchers, epidemiologists, and disease surveillance analysts, who require integrated access to multiple One Health data streams and interactive modeling capabilities. This framework helps them model and understand disease dynamics using integrated One Health data. The system combines data from human clinical records, environmental monitoring, and wastewater surveillance into a single, unified platform. Central to this system is a mixed-effects statistical modeling engine that enables predictive modeling while accounting for variability across different counties, time periods, and population groups. The models are implemented in Python 3.12 using the statsmodels library (MixedLM), with parameters estimated using restricted maximum likelihood (REML). This formulation supports flexible specification of fixed effects for predictors and random effects for grouping variables, allowing the model to capture hierarchical and cross-sectional variability in the data.

Our framework is designed to help domain experts iteratively build and evaluate models, explore trends, and make evidence-based decisions. Unlike many traditional tools, the system offers interactive features that allow users to upload datasets, select predictors, visualize trends, compare regions, and configure modeling parameters without coding.

To ensure the reliability and usability of the proposed framework, this work employed a two-tiered evaluation approach encompassing both quantitative validation and qualitative assessment. For quantitative validation, model performance was evaluated using a rolling time-series cross-validation framework, in which models were trained and tested on temporally separated data partitions to assess out-of-sample predictive performance. Performance was quantified using Root Mean Square Error (RMSE), Mean Absolute Error (MAE), and Spearman correlation across multiple counties and outcome variables. To assess model robustness across time, these metrics were computed across multiple validation folds, providing an indication of performance stability under different temporal splits.

The validation stage was conducted independently from exploratory modeling steps used for feature selection and hypothesis generation, thereby reducing the risk of optimistic bias. Qualitatively, the system was evaluated through expert feedback sessions involving disease surveillance analysts and epidemiologists, focusing on interpretability, workflow integration, and cognitive workload. Using the NASA Task Load Index (NASA-TLX), a widely used subjective workload assessment tool that measures perceived cognitive and physical demands across multiple dimensions (e.g., mental demand, effort, and frustration) [19], along with open-ended interviews, participants assessed the usability and mental demand of the tool’s interactive modeling and provenance-tracking features. Together, these results indicate that the system produces stable predictive performance under cross-validation while supporting user-centered analytical reasoning and model comparison within public health workflows.

Overall, this work makes three contributions. First, we present OH-MEMA, an integrated visual analytics workflow that unifies data preparation, interpretable mixed-effects modeling, model comparison, and analytic provenance for One Health surveillance. Second, we operationalize a lag-aware mixed-effects modeling pipeline for heterogeneous county-level time series, combining the STL-based signal extraction, nonlinear fixed effects, and flexible random-effects specification within a no-code interface. Third, we evaluate the framework through rolling time-series prediction experiments and a formative expert usability study with public-health users. The overall contribution lies in integrating established statistical components into a transparent, reproducible, human-in-the-loop analytical system.

## 2. Related Work

Understanding how our framework fits within the broader landscape of visual analytics and epidemiological modeling requires examining several interconnected research areas. Reviewing these domains highlights gaps in existing systems and motivates our design for a unified, interpretable, and interactive modeling workflow across human, animal, and environmental health data.

### 2.1. Visual Analytics for Health and Epidemiology

Modern visual analytics has evolved beyond simply creating attractive charts and focuses on supporting human reasoning throughout the entire analytical process [20,21]. In health applications, this means helping domain experts explore data, build models, and validate findings in ways that leverage their expertise while benefiting from computational power. For instance, Bryan et al. [22] developed spatiotemporal forecasting systems that integrate predictive analytics with geographic simulation, demonstrating that visual tools can support real-time decision-making during the pandemic. However, significant challenges remain. During health emergencies, many visualization tools fail to align with users’ actual needs, often contributing to cognitive overload rather than supporting clear decision-making [10].

### 2.2. Mixed-Effects Modeling and Machine Learning Integration

Our framework combines traditional statistical approaches with modern computational methods. Visual analytics can enhance every stage of the machine learning pipeline, from data quality assessment to model interpretation, which is particularly important for mixed-effects models where understanding both population-level and group-specific patterns is crucial [23].

While recent work has demonstrated the feasibility of integrating random effects into complex machine-learning models, such as gradient-boosting frameworks [24], our system intentionally adopts a statistical mixed-effects model with a nonlinear transformed predictors approach. This design choice reflects a trade-off between predictive flexibility and the requirements of interpretability, stability, and usability in public health decision-making. The mixed-effects model with nonlinear transformed predictors provides explicit representations of fixed effects, random effects, and temporal lag structures, enabling domain experts to directly inspect effect sizes, uncertainty, and cross-county or demographic variability. In contrast, more complex ML-based models often introduce higher model opacity, require extensive hyperparameter tuning, and incur increased computational overhead, which can hinder interactive exploration and real-time model iteration. Moreover, mixed-effects models are well-suited to epidemiological settings characterized by limited sample sizes, missing data, and heterogeneous reporting practices, offering stable estimation and straightforward deployment within operational surveillance workflows. These properties make the mixed-effects model with nonlinear transformed predictors a natural fit for OH-MEMA’s visual, human-in-the-loop analytical paradigm.

The integration of feature selection with mixed-effects approaches has also advanced significantly. Visual analytics platforms, such as FeatureEnVi [25], combine automated feature selection with user-guided exploration. This interactive feature engineering enables domain experts to leverage their knowledge while benefiting from algorithmic optimization. This approach is particularly beneficial in One Health datasets that encompass diverse variables with complex relationships.

### 2.3. Interactive Systems for Epidemiological Modeling

Modern epidemiological modeling increasingly emphasizes collaboration between human experts and computational tools. Stolper et al. [26] pioneered progressive visual analytics approaches that enable users to interact with algorithms as they operate, allowing users to adjust the analysis direction based on intermediate results. This concept has yielded valuable insights in health applications, where model assumptions may need to be rapidly adjusted as new information becomes available. Makonin et al. [27] extended this work by proposing mixed-initiative systems where both users and algorithms take turns leading the analysis. Their framework includes intelligent data preparation, automatic visual suggestions, and system-guided exploration while maintaining user control. Several successful applications demonstrate the potential of these approaches. Reinert et al. [11] developed PanViz 2.0 as a pandemic modeling tool that integrates epidemiological simulation with interactive visualization, demonstrating how public health decision-makers can evaluate intervention strategies through visual interfaces. The collaborative approach is also reflected in systems like TimeFork [28] and frameworks based on “opposite models” [29], where users guide model construction through interactive visual interfaces. The current study employs this approach to building mixed-effects models.

### 2.4. One Health Data Integration and Visualization

The One Health paradigm presents unique challenges for visual analytics. One major challenge is in integrating human clinical data with environmental and animal surveillance information at appropriate spatial and temporal scales [3,4,5]. For instance, Mukherjee et al. [5] investigated methods for integrating veterinary syndromic data with human health indicators, demonstrating the potential of cross-species data integration for enhanced disease surveillance. However, many existing systems treat different data sources as separate analytical streams rather than truly integrated inputs to unified models [1,30]. Our framework addresses this gap by incorporating One Health data sources directly into mixed-effects modeling workflows.

### 2.5. Evaluation and Validation in Health Visual Analytics

Rigorous evaluation remains a critical challenge in health visual analytics [10,14]. The ESID tool  [31] identifies common evaluation gaps, including insufficient attention to real-world usability and limited assessment of decision support effectiveness. Recent work has begun developing more robust evaluation approaches. Rydow et al. [32] introduced visual sensitivity analysis methods that help researchers understand how parameter variations influence model outputs, directly relevant to our mixed-effects modeling context. Xu et al. [13] developed a platform that utilizes advanced visualizations, including textured-tile calendars and multivariate rose charts, to compare model predictions with actual data across space and time. Case studies reveal that the platform can uncover prediction uncertainty error trends in barely populated areas.

### 2.6. Positioning Our Contribution

Our framework addresses several key gaps identified in these works. While existing visual analytics approaches have demonstrated value in epidemiological contexts, few systems effectively integrate One Health data sources within unified analytical workflows. Current mixed-effects modeling tools often lack the interactive capabilities needed for rapid model development and validation during health emergencies. Most importantly, existing approaches rarely combine the interpretability of traditional statistical methods with the interactive capabilities needed for practical public health decision-making.

A key innovation of our system is the visual analytics provenance tree, which captures the evolution of modeling configurations and associated performance metrics over time. This tree allows users to track and compare runs based on selected predictors (e.g., wastewater SARS-CoV-2 concentrations, the Net Effective Temperature (NET)), outcome variables (e.g., hospitalizations), and random effects (e.g., county, month, age group). Each node in the tree encodes evaluation metrics such as RMSE, MAE, and correlation through color and size, enabling intuitive visual comparison. This design promotes transparency, supports iterative refinement, and encourages evidence-based model selection.

Beyond this visualization component, our system introduces a set of interactive capabilities that enable rapid model development and exploration. Users can select outcome variables and input features through a guided interface, configure random effects using dropdown menus, and initiate model training with a single click. Model training is handled server-side and optimized for fast computation, returning results within seconds for datasets at a weekly level. Once models are built, users can drill down into specific configurations, view comparative performance, and assess how different modeling choices impact predictive accuracy. These capabilities lower the barrier to entry for domain experts without statistical training, while still supporting advanced analyses for public health researchers and epidemiologists.

By combining explainable modeling techniques with interactive controls, performance feedback, and provenance tracking, our framework enables a more agile, user-centered modeling workflow. It empowers stakeholders to quickly explore multiple hypotheses, refine models in real time, and uncover insights that would be difficult to obtain with traditional static modeling tools. In doing so, our system advances the state of the art in visual analytics for syndromic surveillance and demonstrates the practical value of integrating One Health data for timely public health response.

## 3. Materials and Methods

Our goal was to support data-driven insights into pandemic prediction at the county level through a transparent modeling process. This process integrated data-driven feature selection with a mixed-effects model with nonlinear transformed predictors, accounting for human clinical outcomes, environmental conditions, and wastewater-based epidemiology.

### 3.1. Deployment and Computational Considerations

OH-MEMA is a lightweight, white-box visual analytics tool with a modular client–server architecture that supports interactive visual analytics and iterative model exploration. Input datasets are uploaded as structured CSV files and processed in-memory during a user session; uploaded data are not persistently stored or written to a database. This design supports privacy-preserving analysis and deployment in sensitive public health contexts.

A preprocessing layer performs temporal alignment, missing value handling, normalization, and time-series decomposition prior to analysis. Statistical modeling is implemented in a backend analytics engine that supports mixed-effects regression, lagged predictor selection, and rolling time-series validation. Model configurations and results are cached only for the duration of the session to facilitate comparison across analytical iterations.

The visualization layer is implemented as a web-based interactive interface that provides coordinated views, including time-series charts, correlation heatmaps, model comparison panels, and analytics provenance trees. Communication between the analytics backend and the visualization frontend occurs through asynchronous API calls, enabling responsive user interaction. The modular design allows the system to be extended with additional data sources, modeling approaches, or visualization components without modifying the core workflow.

OH-MEMA is designed for flexible deployment in on-premises, cloud-based, or hybrid environments. Because uploaded datasets are processed in-memory and not persisted, the system can be deployed in privacy-sensitive institutional settings or on cloud platforms to support remote access and collaborative analysis. This flexibility ensures that OH-MEMA can be adapted to diverse operational contexts while maintaining data security and user privacy.

Computational requirements are modest due to the lightweight design. For county-level datasets similar to those used in our case studies, all preprocessing, including missing value handling, normalization, and STL decomposition, as well as model fitting and visualization, can be completed in just a few seconds on a modern laptop or desktop. Anticipated server workload scales with the number of users and dataset size, but the modular architecture allows deployment on multi-core servers or cloud platforms to support multiple concurrent sessions while maintaining responsiveness. OH-MEMA also supports parallel computation. Within a session, different counties or model configurations can be processed concurrently. Multiple users can run isolated sessions simultaneously without interference. For larger datasets or higher-concurrency scenarios, deploying on multi-core servers or cloud platforms ensures continued responsiveness and scalability. This design makes OH-MEMA efficient, interpretable, and suitable for multi-user public health analytics workflows.

**Figure 1:**
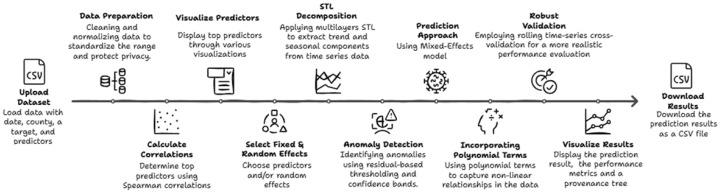
Data Analysis and Modeling Process.

As shown in Figure 1, we began by preparing the dataset for modeling the target variable and its predictors through data cleaning and normalization. Missing values were handled through a combination of interpolation and mean imputation, depending on gap length [33,34]. Short gaps of up to two consecutive weeks were filled using linear interpolation to preserve local temporal continuity, while longer gaps were imputed using county-specific mean values computed within the same seasonal period to avoid introducing artificial trends. This threshold reflects the weekly resolution of the data and common practices in epidemiological time-series analysis.

To evaluate the robustness of this approach, we conducted sensitivity analyses comparing three strategies: (1) our primary method (interpolation for short gaps + seasonal mean for longer gaps), (2) interpolation-only, and (3) complete-case analysis excluding missing weeks. Results showed that the mixed-effects model with nonlinear transformed predictors and estimated effect sizes were largely consistent across methods, with negligible changes in RMSE and MAE for the target variables. These findings support the robustness of our framework and justify the chosen imputation strategy, which balances temporal continuity preservation with avoidance of artificial seasonal distortions.

Following imputation, Min–Max normalization was applied to scale predictors to the [0, 1] range, improving numerical stability and comparability across heterogeneous variables [35]. This approach was chosen because OH-MEMA integrates modeling with visual analytics, and Min–Max normalization allows consistent scaling across predictors, facilitating intuitive visual comparison and interactive exploration. While Min–Max scaling can alter the absolute magnitude of regression coefficients in mixed-effects models with nonlinear transformed predictors, it preserves the relative relationships between predictors, ensuring that effect sizes remain interpretable in terms of relative influence. Furthermore, normalization reduces the exposure of absolute values when combined with aggregation and controlled data access [36], supporting secure and privacy-conscious analyses.

Subsequently, Seasonal-Trend Decomposition using LOESS (STL) [37] was applied independently to each county to extract trend and seasonal components while preserving spatial heterogeneity, enabling the model to capture both short-term fluctuations and seasonal patterns in epidemiological time series. The statistical motivation for STL decomposition is to separate long-term trends and seasonal cycles from short-term fluctuations, reducing noise and improving the stability and interpretability of a mixed-effects model with nonlinear transformed predictors. By decomposing the time series, the model can focus on meaningful temporal patterns without being overly influenced by transient spikes or reporting variability.

To evaluate the effect of STL preprocessing on model robustness, we compared model fitting on STL-processed versus raw series. These comparisons guided preprocessing choices and ensured that the decomposition did not distort temporal relationships or introduce bias. Detailed evaluation of predictive performance with and without STL decomposition is presented in the Results Section.

To enhance the interpretability and modeling capabilities of epidemiological predictors in our visual analytics system, we employed a layered STL to decompose and denoise the county-level time series [37]. The process began with a log transformation to stabilize variance and reduce the impact of extreme values, which was particularly relevant for count-based health metrics [38]. We then decomposed the target variable and each predictor using STL and compiled a list of cleaned, county-specific decomposition results. This list included the trend, multiple seasonal layers (yearly, quarterly, monthly), and residual components.

To support anomaly detection and interpretation in the visual interface, we employed a residual-based thresholding mechanism grounded in rolling statistics. A centered moving average and residual-based thresholds were computed over a configurable window applied to the final residuals from the multi-layer STL decomposition. Observations exceeding these thresholds were flagged as anomalies, indicating potential outbreaks or shifts in the signal, and corresponding upper and lower confidence bands were retained for visual encoding. This approach resembles Bollinger Bands [39], a widely used technique for time-series anomaly detection, and aligns with established statistical process control practices in public health surveillance [40].

**Figure 2:**
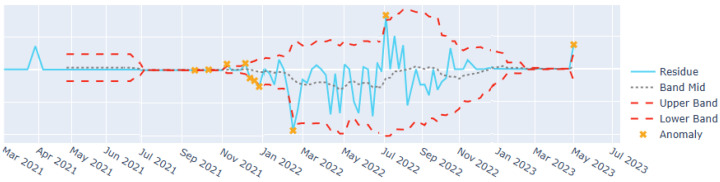
Anomaly detection from STL decomposition showing residual, band mid, upper band, lower band, and anomalies.

To improve robustness to non-stationary variance, the rolling window adapts to local residual fluctuations, reducing sensitivity to short-term spikes. Sensitivity analyses using synthetic and historical outbreak events confirmed that the procedure highlights meaningful deviations while maintaining a low false positive rate. Anomaly flags and confidence bands were retained as first-class data attributes and visually encoded to support interactive identification of atypical patterns and emerging signals, as illustrated in Figure 2.

After that, we updated the model preparation pipeline to conditionally include random effects and random slopes in the final modeling DataFrame. The “month” column was excluded from the “date” column to be used as a random effect. This ensured compatibility with models that expect these variables and resolved errors related to categorical variable aggregation and unintended duplication.

The prediction approach builds on that of Daza-Torres et al. [17], who also used a mixed-effects model for prediction. However, we extend their methodology in several significant ways. While their research focuses exclusively on COVID-19 hospitalization as the disease outcome, our model is designed to predict a variety of pandemic outcomes, including cases, deaths, and hospitalizations. We have broadened the set of predictors from the original focus on COVID-19-related data, such as wastewater, test positivity rate (TPR), and reported cases, to include additional data sources, including environmental factors like the Net Effective Temperature (NET) index, as well as other relevant predictors. Furthermore, whereas their analysis models the interaction between wastewater and the COVID-19 wave, our approach accommodates interactions between any predictor and other temporal factors.

We also incorporate polynomial terms to capture non-linear relationships [41]. For each county, continuous predictors (e.g., weather indicators) are log-transformed and expanded with cubic polynomial terms. Both continuous and binary anomaly features (e.g., weather anomalies) are shifted across a defined temporal window of lags (from −4 to 0 weeks), enabling systematic testing of lead–lag relationships between predictors and the target variable [42]. To mitigate overfitting risks associated with multiple polynomial expansions and lagged predictors in relatively short time series, we adopt several safeguards: (1) lag selection is performed separately within each cross-validation fold, ensuring that information from the evaluation period is not used for selection and avoiding potential leakage [43], (2) polynomial order is limited to cubic to prevent excessive flexibility, and (3) predictors are retained only if they consistently demonstrate meaningful temporal associations across folds. This approach balances capturing non-linear dynamics with maintaining model stability, interpretability, and unbiased assessment of predictive performance.

Additionally, we have expanded the random effects structure from a single grouping by county to a more comprehensive framework that accounts for variability by age group, sex group ratios, and temporal effects across months.

**Figure 3:**
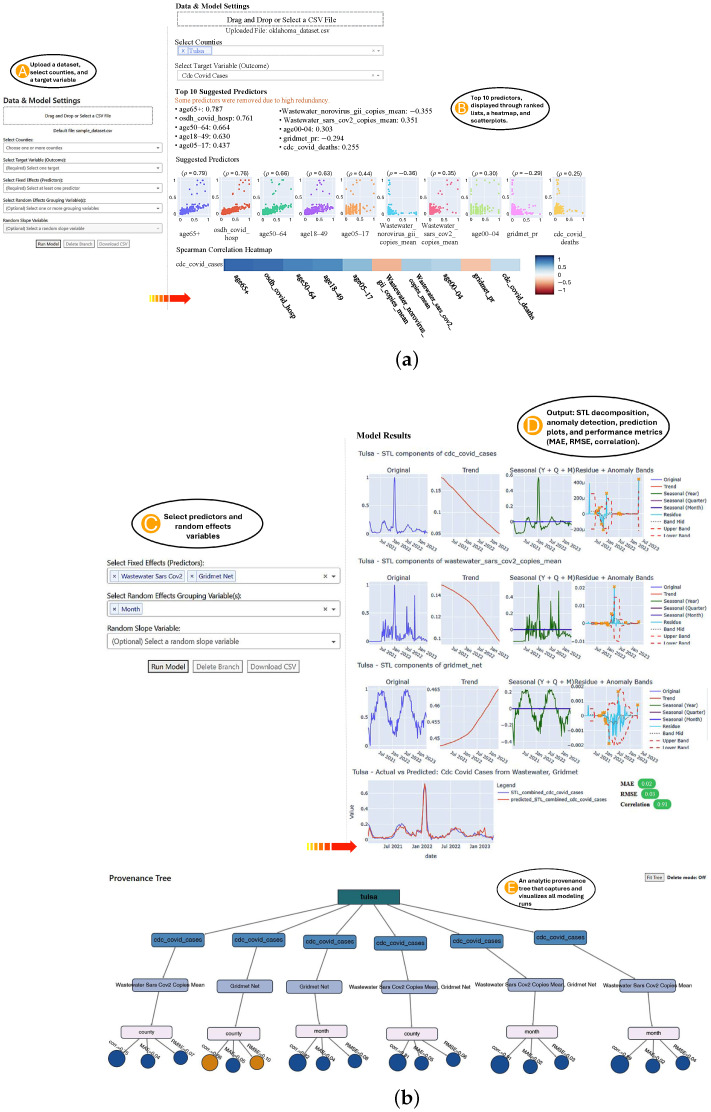
Visual Analytics Workflow for Polynomial Lagged Mixed-Effects Modeling. (**a**) Data upload, target selection, and predictor exploration: (**A**) After uploading a dataset, users select counties and a target variable. (**B**) The system computes Spearman correlations between the target and all other variables and presents the top predictors using ranked lists, a heatmap, and scatter plots. (**b**) Model specification, output visualization, and analytic provenance. (**C**) Users configure fixed and random effects, optionally specify interaction (random slope) variables, and run the model. (**D**) Output includes STL decomposition, anomaly detection, prediction plots, and model performance metrics (MAE, RMSE, correlation). (**E**) An analytic provenance tree captures and visualizes all modeling runs, enabling users to explore past configurations, compare performance, and reload previous models. Larger blue circles indicate stronger correlations or lower errors than smaller blue circles. The system is available at https://mema.disc.ourcloud.ou.edu/ (accessed on 1 March 2026).

Importantly, our approach is interactive, allowing users to choose their outcome, predictors, and grouping structures through a visual tool that highlights key relationships and provides real-time results, as shown in Figure 3.

Finally, unlike the original study, which reports results from a single, static model fit, our method utilizes rolling time-series cross-validation for each county. This yields a more robust and realistic evaluation of predictive performance.

### 3.2. Model Formulation

We model the log-transformed outcome using a lag-aware mixed-effects specification that combines transformed fixed effects with optional random effects. For county *i* at time *t*, the general model is

(1) $$y_{it}=\beta_{0}+\sum_{p=1}^{P}\beta_{p}\;\phi_{p}\;\left(x_{p,i,t-l_{p}}\right)+\sum_{q=1}^{Q}\delta_{q}z_{qit}+\sum_{h=1}^{H}b_{g_{h}\left(i,t\right)}^{\left(h\right)}+u_{g_{r}\left(i,t\right)}^{\left(r\right)}\;\phi_{r}\;\left(x_{r,i,t-l_{r}}\right)+\epsilon_{it},$$

where $y_{it}$ denotes the log-transformed target outcome (e.g., COVID-19 hospitalizations) for county *i* at time *t*, and $\beta_{0}$ is the global intercept. The term $x_{p,i,t-l_{p}}$ denotes predictor *p* observed at lag $l_{p}$, and $\phi_{p}\left(\cdot \right)$ denotes the transformation applied to that predictor, such as the identity function or a user-specified polynomial expansion. The coefficient $\beta_{p}$ is the corresponding fixed-effect parameter. Additional fixed-effect covariates, including interaction terms or anomaly indicators, are denoted by $z_{qit}$ with coefficients $\delta_{q}$. For each candidate predictor, the lag $l_{p}$ is selected from a pre-specified set of allowable lags L defined by the analyst. To avoid information leakage, lag selection is treated as a model-selection step and is performed using only the training portion of each rolling validation split; the selected lag is then carried forward to the corresponding held-out test fold. Thus, the reported predictive performance reflects out-of-sample evaluation rather than lag selection on the full dataset. The term $b_{g_{h}\left(i,t\right)}^{\left(h\right)}$ denotes a random intercept for the grouping factor $g_{h}$, where candidate grouping factors include county, month, age group, and sex group. When multiple grouping factors are included in the same model, they are treated as crossed random effects unless otherwise stated. The term $u_{g_{r}\left(i,t\right)}^{\left(r\right)}\;\phi_{r}\;\left(x_{r,i,t-l_{r}}\right)$ represents an optional random slope that allows the effect of a selected predictor $x_{r}$ to vary across a chosen grouping factor. Finally, $\epsilon_{it}∼N\left(0,\sigma^{2}\right)$ denotes the residual error term. This formulation is intended for interpretable predictive modeling of temporal associations and hierarchical variation in surveillance data. It does not support causal interpretation of the relationships between environmental predictors and clinical outcomes.

### 3.3. Model Specification, Software, and Estimation

All models were fitted in Python using the statsmodels MixedLM implementation (version 0.14.4) with restricted maximum likelihood (REML). The OH-MEMA interface records the fixed-effects formula, predictor transformations, selected lag(s), and random-effects structure for each model run. In the case studies reported here, county, month, age group, and sex group were evaluated both as alternative single random-effects specifications and, in combined configurations such as C + M + S and C + M + A, as joint random-effects structures with county used as the primary grouping factor and the remaining factors included as additional variance components. Continuous predictors were modeled on the log-transformed scale and, for selected continuous effects, expanded with cubic terms to capture nonlinearity. Model fitting used the default optimizer sequence provided by statsmodels MixedLM with the package’s default convergence settings. No optimizer-specific tolerances were manually overridden in the current implementation. If a candidate predictor–lag combination or final county-specific model failed during fitting, that specification was skipped and excluded from model comparison. Hyperparameters and model-specification choices, feature selection via visual analytics, user-configurable mixed-effects modeling, and explainable evaluations of the results are all delivered through an online, interactive tool, responding to held-out fold. Unless otherwise noted, coefficient estimates are summarized with standard errors, and predictive performance is summarized across validation folds.

### 3.4. Random Effects Handling

To account for variability across spatial and demographic dimensions, we incorporated random effects into our mixed-effects modeling framework. Random effects enable the model to capture unobserved heterogeneity that arises from grouping structures, such as geography (e.g., counties), time (e.g., months extracted from the date), or population characteristics (e.g., age or sex groups). This approach aligns with previous research that emphasizes the importance of hierarchical structure in public health data modeling [24].

### 3.5. Prediction Model Evaluation

To ensure robust performance estimation, we implement rolling time-series cross-validation for each county. The procedure is as follows:

- The time series for each county is split chronologically, with the earliest 80% of observations used for initial training and the most recent 20% reserved for testing, simulating a realistic forecasting scenario.
- The training set expands forward in time, and the model is retrained at each step (forward-chaining CV), creating multiple rolling folds. For our dataset, this resulted in 5 rolling folds, balancing sufficient training data for reliable model estimation with adequate coverage of the test period.
- The forecast horizon is 1 week, reflecting the weekly temporal resolution of the data and the practical requirements of county-level forecasting.
- Lagged versions of key predictors (e.g., NET, wastewater concentrations) are tested independently within each training fold, and the best lag is selected using only the training data, avoiding information leakage from the test set.
- All model hyperparameters, including polynomial order and random effect structure, are re-estimated separately within each training fold to ensure unbiased performance evaluation.
- Model performance is evaluated on the held-out test set for each fold using mean absolute error (MAE), root mean squared error (RMSE), Spearman correlation, and $R^{2}$, and metrics are averaged across folds to summarize predictive accuracy.

This procedure provides a realistic, temporally consistent evaluation of predictive performance, accounts for non-stationary time series patterns, and avoids information leakage between training and testing. The number of folds and forecast horizon were chosen to ensure that each fold contains sufficient observations for stable mixed-effects model estimation while enabling multiple evaluation periods for robust assessment.

## 4. Visual Analytics System Description

Our methodology is based on three main pillars: selecting features through visual analytics, user-configurable mixed-effects modeling, and providing explainable evaluations of the results, all delivered through an online interactive tool as shown in Figure 3. The full system is deployed and freely accessible via a web interface at https://mema.disc.ourcloud.ou.edu/ (accessed on 1 March 2026). Users can utilize the provided sample dataset or upload their own dataset to view the results quickly and easily.

### 4.1. Data Preparation and Input Requirements

OH-MEMA is designed to accept structured, tabular time-series data in CSV format. Each dataset must include a temporal attribute (i.e., date), a spatial identifier (e.g., county), and at least one numeric outcome variable to be predicted, along with one or more numeric predictor variables. All datasets are aligned to a common temporal resolution, which in our case studies is weekly, to ensure consistency across human, environmental, and wastewater data sources.

### 4.2. Feature Selection

Once the user uploads a CSV file containing relevant data, such as county, date, and at least one outcome variable, the system identifies valid predictors and outcomes. When the user selects a target variable (for example, COVID-19 hospitalizations), as shown in Figure 3A, the system calculates Spearman correlations between this target and all other columns in the dataset.

To improve interpretability while preserving multivariable predictive potential, the system implements a two-step heuristic with safeguards against multicollinearity:

1. Correlation ranking: Predictors are ranked by absolute correlation with the target to prioritize candidates for visual exploration. This ranking is a lightweight guide for users rather than a strict selection criterion; final inclusion is determined by multivariable model evaluation.
2. Redundancy filtering: Predictors with pairwise correlation above a configurable threshold (e.g., 0.95) are flagged, and only one representative feature from each highly correlated group is suggested for inclusion. Users are notified of removed predictors.

Additional safeguards ensure stability when including polynomial expansions and lagged predictors:

- Polynomial terms are limited (e.g., cubic) to avoid excessive redundancy from higher-order terms.
- Lagged predictors are tested individually, and only the most relevant lags are retained through cross-validated selection.
- The mixed-effects model with nonlinear transformed predictors framework accounts for remaining collinearity by partitioning variance between fixed and random effects, maintaining stable coefficient estimates.

After this preprocessing, the top non-redundant predictors are presented via a ranked list, interactive scatterplots, and a correlation heatmap, as shown in Figure 3B. This selection process, summarized in Algorithm 1, guided user involvement by combining statistical associations with visual pattern recognition. Users can select one or more predictors to include as fixed effects and specify variables such as county, month, age, or sex group as random effects. Interaction terms between a selected slope variable and random effects can also be specified. Once selections are made, the user clicks the “Run Model” button to perform calculations, as shown in Figure 3C.

This approach balances visual and statistical guidance with user control, reduces redundancy, and ensures that multivariable predictive performance, rather than univariate correlation alone, drives the final model.

### 4.3. User Interface

Our system’s user interface is built to support transparency, user control, and step-by-step interaction for non-expert users in public health and epidemiology. It consists of four main panels, as shown in Figure 3.

- Panel A (Data Selection): Users can upload a CSV file or use the sample dataset that is included in the system. The interface automatically validates column names and updates the drop-down menus accordingly.
- Panel B (Feature Correlation View): The system computes Spearman correlations between the selected target and all predictors. The predictors that are highly correlated with each other (above a configurable threshold, e.g., 0.95) are automatically filtered, leaving only one representative feature from each correlated group. Users are notified when predictors are removed due to redundancy. The top 10 predictors are displayed in a ranked list, interactive scatter plots, and a correlation heatmap.
- Panel C (Model Configuration): Users configure fixed and random effects. Optional controls allow the selection of interaction terms and slope variables. Tooltips explain the statistical meaning of each option, helping non-expert users understand mixed-effects modeling decisions
- Panel D (Results & Interpretation): Model predictions, STL decomposition results, and detected anomalies are shown with overlaid line charts. Evaluation metrics (MAE, RMSE, and correlation) are displayed, and results can be downloaded as a CSV file.
- Panel E (Version History and Model Traceability): Every model run is automatically stored with its configuration, selected county, target, predictors, random effects, and its resulting performance metrics. A visual tree interface allows users to explore and compare past runs. Each node displays a modeling decision (e.g., selected predictors or random effects), while performance metrics (correlation, MAE, and RMSE) are shown as color-coded and size-coded leaf nodes. Users can hover over a node to visually highlight it, enabling quick identification within dense trees. Users can click any node to reload a previous configuration, enabling rapid iteration, insight tracking, and reproducible modeling workflows. Users can also delete the unwanted branches by clicking on the “Delete Branch” button and then selecting the node to delete its dependencies. This visual traceability supports iterative exploration, reproducibility, and transparent reasoning.

**Algorithm 1:** Feature Selection and Modeling Workflow  **Input:** CSV file with weekly county-level data  **Output:** The prediction results from the mixed-effects model
  1Step 1: Upload the dataset with columns such as date, county, target, and potential predictors  2Step 2: Select one or more counties to subset the dataset  3Step 3: Choose a target variable  4    •  Spearman correlations will be calculated between the target and all other columns to find the top 10 related predictors  5    •  Visualize the top 10 predictors using:  6     •  Ranked list of correlation coefficients  7     •  Correlation heatmap  8     •  Interactive scatterplots  9Step 4: Select one or more predictors as fixed effects10Step 5 (Optional): Select one or more random effects (e.g., county, month, age group)11Step 6 (Optional): Choose one fixed-effect predictor to model random slopes12Step 7: Run the model and return:13    •  STL decomposition of predictors and target14    •  Anomaly detection15    •  Model prediction results with performance metrics (MAE, RMSE, correlation)16    •  Append current model configuration and metrics to an analytics provenance tree for visualization17Step 8 (Optional): Delete a branch from the analytics provenance tree18    •  The delete button is enabled only after at least one model run has produced a tree19    •  Selecting a branch allows the user to remove that branch and all its associated model configurations from the tree20    •  If the selected branch is the only branch in the tree, the system prompts the user for confirmation before deletion

The interface is implemented using Dash 4.0 and Plotly 6.6 for interactive visualization, enabling dynamic updates as users modify their selections. Tooltips, hover info, and dropdown menus provide further guidance during exploration. This interface acts as a white-box system, promoting transparency and explainability in model building.

### 4.4. Analytics Provenance for Model Exploration and Comparison

To support iterative model refinement and comparative analysis, we integrated an analytics provenance mechanism [44] into the dashboard, as shown in Figure 3E. Each time a model is run, the system logs the full configuration, including the county, target variable, fixed-effect predictors, random-effect groups, polynomial orders, selected lags, and other model hyperparameters, along with the resulting evaluation metrics (correlation, MAE, and RMSE). These logged versions are visualized as a dynamic, interactive tree using Dash Cytoscape in Python, where each branch represents a modeling decision (e.g., target variable, predictors, random effects), and leaf nodes display the corresponding performance metrics.

The use of provenance trees is grounded in extensive research demonstrating their value for tracking analytic workflows and supporting sensemaking in complex data analysis tasks [45,46]. Provenance visualization captures the history and rationale behind analytic decisions, enabling users to retrace steps, compare alternative hypotheses, and understand how changes in model parameters impact outcomes [47,48]. By explicitly logging all model parameters, the system ensures reproducibility, allowing users to exactly replicate previous modeling runs and validate results. This explicit representation of analytic provenance is particularly critical in epidemiological modeling, where transparency and reproducibility are necessary for building trust with domain experts and decision-makers [49].

To improve interpretability, leaf nodes are colored using a color-blind-safe palette [50] to provide quick insight into model quality: blue indicates strong correlation (ρ>0.7) or low error (MAE/RMSE <0.1), while orange highlights weaker performance. Node size encodes the magnitude of performance, with larger nodes corresponding to stronger correlations or lower errors. These visual encodings help users quickly identify optimal models and support inclusive, feedback-driven exploration.

The tree structure supports an overview and detailed analytical workflow, enabling users to quickly evaluate model performance across multiple experiments and identify effective configurations, such as those with low MAE or high correlation coefficients. Users can interactively reload previous runs by clicking on any node, facilitating iterative exploration and model refinement. This interactivity aligns with best practices in provenance visualization research, which emphasizes user-driven exploration of analytic histories to support trade-off analysis and informed decision-making [51,52].

## 5. Case Study: County-Level Surveillance in Oklahoma (2020–2024)

To assess the effectiveness of our system in supporting proactive public health interventions and understanding outbreaks, we focus on a real-world case study in the state of Oklahoma. This study integrates geospatial and temporal datasets across both human and environmental health domains, in line with the One Health approach. Our objective is to explore correlations and predictive relationships among various data sources at the county level from 2020 to 2024. The case study encompasses all 77 counties in Oklahoma, with data aggregated weekly for the human clinical, wastewater, and environmental datasets.

- Human Clinical Data from CDC and OSDH: We incorporate aggregated weekly-level COVID-19 clinical data, including cumulative confirmed cases, hospitalizations, and deaths. These records are sourced from both the U.S. Centers for Disease Control and Prevention (CDC), which collects standardized data across all states and territories [53], and the Oklahoma State Department of Health (OSDH), which provides more localized reporting. These outcome variables serve as core targets for predictive modeling and anomaly detection.
- Wastewater Surveillance of Human Pathogens: This dataset includes quantifications of human pathogens, such as SARS-CoV-2, Influenza, and Respiratory Syncytial Virus (RSV), from municipal wastewater samples collected across Oklahoma counties. Wastewater surveillance offers a non-invasive, early-warning signal of community-level pathogen presence, even when clinical testing is underreported or delayed [54].
- Environmental and Meteorological Data (gridMET): We include environmental data from the gridMET dataset, which provides high-resolution gridded daily surface meteorology for the contiguous United States. Variables such as minimum and maximum temperatures, wind speeds, vapor pressure deficits (VPD), and relative humidities are aggregated at the county level [55]. To quantify thermal comfort conditions at the county level, we compute the Net Effective Temperature (NET) index, an indicator that accounts for the combined effects of air temperature, relative humidity, and wind speed. The NET index [56], is defined as
  (2) $$\mathrm{NET}=37-\frac{37-T}{0.68-0.0014\cdot RH+\frac{1}{1.76+1.4\cdot v^{0.75}}}-0.29\cdot T\cdot \left(1-0.01\cdot RH\right)$$
  where *T* is the daily mean near-surface air temperature (in °C), RH is the relative humidity (in %), and *v* is the wind speed (in m/s). NET provides a more holistic representation of thermal stress than temperature alone and has been widely applied across diverse regions using established thermal comfort classification schemes [56]. Prior work defines thermally comfortable conditions at intermediate NET values (approximately 9–23 °C), with thermal discomfort increasing at both lower and higher extremes [56]. In our case study, we calculate weekly mean NET values from gridMET meteorological data for each Oklahoma county, which serve as a key environmental predictor of respiratory-related health outcomes. We hypothesize that deviations from thermally comfortable conditions, corresponding to low or high NET values, are associated with increased COVID-19 transmission, potentially due to behavioral responses such as increased time spent indoors under thermally stressful conditions. This expected non-linear relationship motivates our use of polynomial terms when modeling the effect of NET.

Table 1 summarizes the Oklahoma case-study dataset used in both analyses, including the geographic scope, temporal coverage, aligned data sources, and outcomes considered. These case studies demonstrate feasibility and practical utility within one state-level surveillance context; they do not establish external generalizability across regions or diseases.

### 5.1. Case Study I

As a case study, we apply our visual analytics framework to explore the correlation between multiple heterogeneous datasets and their predictive power for COVID-19 hospitalizations in Tulsa County, Oklahoma. The goal is to support proactive public health surveillance by examining the relationship between environmental and wastewater signals and observed trends in human health outcomes.

In our modeling approach, variables derived from wastewater surveillance and environmental datasets are treated as fixed effects variables, representing candidate predictors with consistent associations with the target outcome. The outcome of interest is the weekly number of COVID-19 hospitalizations at the county level. To capture hierarchical and contextual variation, we include random effects for county, month, age group, and sex group. These allow the model to account for unobserved heterogeneity across demographic strata and temporal contexts.

We assessed three predictive models (M1–M3) using results from our interactive visual analytics system, as shown in Table 2. This includes model evaluation metrics (e.g., MAE, RMSE) and correlation statistics, all computed on held-out validation data from the most recent 20% of the time series using rolling time-series cross-validation. These metrics reflect model performance on future observations not included in the training set. To prevent information leakage, all data preprocessing steps, including missing value handling, normalization, STL decomposition, and feature selection based on Spearman correlation, were performed exclusively within the training portion of each rolling split. The validation data were used only for performance evaluation.

To ensure that improvements were not due to information leakage, STL (Seasonal-Trend decomposition using Loess) was applied exclusively within the training portion of each rolling time-series split. Performance gains were consistent across multiple validation folds, indicating that STL captures meaningful temporal structure rather than introducing artificial smoothing effects.

Across all model configurations, incorporating STL led to consistently lower MAE and RMSE, as well as higher Spearman correlation coefficients. Quantitatively, STL-based models achieved substantial and consistent error reductions, with MAE decreasing by approximately 12–44% and RMSE by 10–40%, along with correlation improvements of up to 20 percentage points (Table 2).

For instance, model M3, which combines wastewater and NET predictors, achieved the best overall performance when the random effects included county, month, and age group. This configuration yielded an MAE of **0.067**, an RMSE of **0.093**, and a correlation of **89.47%**, underscoring the value of multi-source integration.

We also observed that the structure of random effects plays a crucial role in prediction accuracy. While models with only a single grouping variable (e.g., county or month) performed reasonably well, those with multiple random effects, particularly combinations such as county, month, and age group, consistently outperformed simpler models. These findings highlight how combining One Health datasets through an explainable and interactive platform can enhance our understanding of outbreak dynamics.

### 5.2. Case Study II

**Figure 4:**
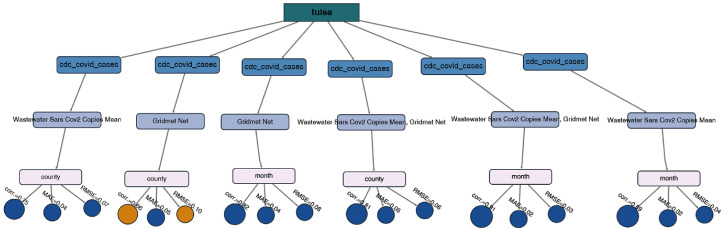
Visual summary of model analytics provenance. Each tree branch represents a unique modeling configuration, including the selected target variable, predictors, random effects, and corresponding model performance metrics. Leaf nodes display correlation (ρ), MAE, and RMSE. Node colors provide quick insight into model quality: blue indicates strong correlation (ρ>0.7) or low error (MAE/RMSE <0.1), while orange highlights weaker performance. Larger blue circles indicate stronger correlations or lower errors than smaller blue circles. This figure complements Table 3 by enabling users to visually trace and compare modeling decisions over time.

**Figure 5:**
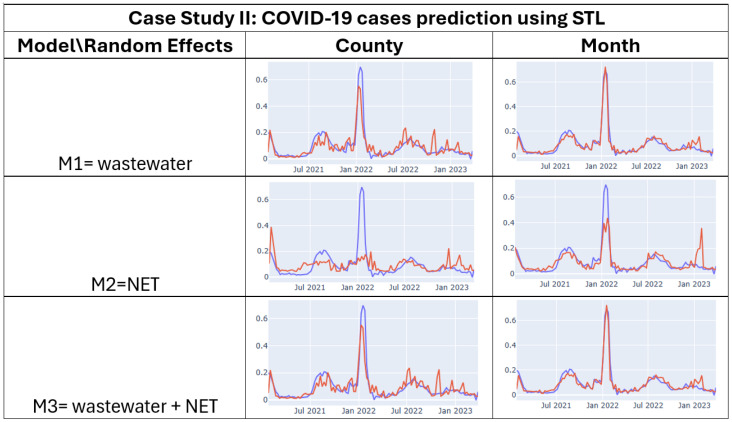
Predicted (red) vs. actual (blue) COVID-19 case counts across selected models. This figure complements Table 3 by visually showing model performance with county or month as random effects.

To evaluate the robustness of our visual analytics system, we conducted a second case study focused on predicting COVID-19 cases. We assessed three models (M1–M3), each incorporating different combinations of predictor types: wastewater SARS-CoV-2 and weather NET index. Table 3 presents the results, showing model performance with and without STL decomposition. Complementing these quantitative results, Figure 4 provides a visual summary of model analytics provenance. Each tree branch represents a unique modeling configuration, displaying the target variable (COVID-19 cases), predictors (wastewater or NET), random effects (county or month), and their associated performance metrics. Leaf nodes show correlation (ρ), MAE, and RMSE, with node colors indicating model quality, blue for strong correlation (ρ>0.7) or low error (MAE/RMSE <0.1), and orange for weaker performance, allowing users to visually trace and compare modeling decisions over time. Figure 5 visualizes the predicted vs. actual COVID-19 case counts across selected models.

Overall, models using STL decomposition consistently outperformed their non-STL counterparts, achieving lower MAE and RMSE and higher Spearman correlation. To ensure that these improvements were not due to chance or information leakage, STL was applied exclusively within the training portion of each rolling time-series cross-validation split, and performance was evaluated on held-out validation data in each fold. This procedure allowed robust assessment of predictive accuracy across time, preventing future information from influencing the training process. The most accurate models included multiple predictors and random effects for the month. For example, the full model (M3), which includes wastewater and NET data, achieved the best performance with STL preprocessing, with an MAE of **0.021**, an RMSE of **0.030**, and a Spearman correlation of **91.28%** across validation folds. By contrast, the same model without STL achieved a median correlation of only **84.28%**. These cross-validated results indicate that STL decomposition captures meaningful temporal patterns and consistently enhances predictive performance rather than introducing artificial smoothing effects.

Interestingly, month-based random effects generally led to stronger model performance than county-based ones, suggesting that seasonal and behavioral patterns are captured effectively across regions. However, we note that this improvement may also partially reflect the relative coarseness of the grouping: since there are fewer months than counties, more data are available per group, which stabilizes random effect estimates and can reduce error [57]. To account for this potential confounding, all model evaluations were performed using rolling time-series cross-validation, comparing county- and month-based random effects on the same held-out validation sets. The consistent improvements observed across multiple folds indicate that month-based random effects capture meaningful temporal structure in addition to benefiting from larger group sizes, providing both robust and interpretable modeling of seasonal dynamics. Among single-source models, wastewater data alone (M1) performed best, followed by NET (M2).

These findings reinforce the benefit of integrating heterogeneous predictors and leveraging STL decomposition to isolate trend and anomaly signals in time-series data. They also demonstrate the system’s utility for facilitating comparative exploration and hypothesis testing across modeling scenarios.

The results from the two case studies demonstrate the potential of our visual analytics framework to support explainable, multivariate, and data-driven public health modeling. By enabling interactive model configuration and displaying clear performance comparisons, the system helps users understand the contribution of different predictors and random effect groupings, promoting transparency and supporting proactive public health decision-making.

## 6. User Evaluation of the OH-MEMA Tool

To assess the usability, effectiveness, and user experience of the OH-MEMA system, we conducted a structured evaluation involving domain experts in public health and disease surveillance in October 2025. This section describes the study design, participation characteristics, procedures, and tasks, as well as the combination of quantitative (NASA-TLX) and qualitative (open-ended) feedback collected. We then present and discuss the evaluation results, highlighting insights into workload, system effectiveness, and user perceptions of interactive modeling and visualization capabilities.

### 6.1. Purpose and Study Design

The purpose of this study was to examine how domain experts interact with the visual analytics interface, interpret model outputs, and integrate mixed-effects modeling into their analytical workflows for syndromic surveillance.

Participants were invited to complete a short 30 to 40 min online session conducted via Zoom. Each session involved hands-on interaction with the OH-MEMA dashboard, where users explored model configuration options, applied mixed-effects modeling, and interpreted resulting visualizations.

### 6.2. Participants and Confidentiality

Participants included two epidemiologists and two disease surveillance analysts. Each was assigned an anonymized ID (e.g., U1, U2). Personal contact information was deleted after scheduling, and all responses were analyzed in aggregate form.

### 6.3. Procedure and Tasks

Each participant was asked to complete a series of guided tasks designed to simulate realistic analytical use of the tool:

1. Select Parameters: Choose a county, a target variable to predict (e.g., COVID-19 cases), and one or more predictors (e.g., NET).
2. Apply Modeling: Run a mixed-effects model using either county or month as a random effect.
3. Explore Results:
  - Compare model performance across different predictor combinations.
  - Interpret results using visual performance metrics.
  - Examine temporal patterns and interdependencies in syndromic surveillance data.
  - Trace and Compare Model Versions:
    – Use the interactive provenance tree to review and compare previously generated models.
    – Identify optimal configurations by inspecting color and size cues that encode model accuracy.
    – Restore prior configurations directly from the tree to continue exploration or refine parameter choices.

After completing the analytical tasks, participants filled out the NASA Task Load Index (NASA-TLX) questionnaire [19] to rate their perceived workload across six dimensions: mental demand, physical demand, temporal demand, performance, effort, and frustration. Each category was briefly explained to participants before rating. Mental demand referred to the amount of cognitive activity required (e.g., thinking, deciding, remembering); physical demand captured the level of physical interaction effort (e.g., mouse or keyboard activity); temporal demand measured the perceived time pressure; performance reflected how successful participants felt in completing the tasks; effort described the overall work required to accomplish the tasks; and frustration represented the degree of stress or irritation experienced during interaction [19]. Each dimension was rated on a 10-point scale (1 = very low, 10 = very high).

### 6.4. NASA-TLX Evaluation

The NASA-TLX instrument was selected as a validated and efficient tool for assessing perceived cognitive and physical workload in interactive analytics environments. Participants provided quantitative ratings on each workload dimension, offering insights into how demanding users found the modeling and visualization processes within OH-MEMA. These measures complemented qualitative feedback to provide a comprehensive and balanced assessment of the user experience.

### 6.5. Open-Ended Feedback

Alongside the NASA-TLX ratings, participants were also invited to provide open-ended feedback in the same online form regarding their perceptions of model effectiveness, usability, and potential for integration:

- The perceived effectiveness of the models they created;
- Features they found confusing or particularly useful;
- The potential integration of the tool into their professional workflow;
- Suggestions for improving usability, clarity, and visualization design.

Responses were qualitatively coded to identify recurring themes related to interpretability, user experience, and analytical efficiency. This analysis provided deeper insight into how users perceived the system’s support for model exploration and decision-making.

### 6.6. User Evaluation Results and Discussion

**Figure 6:**
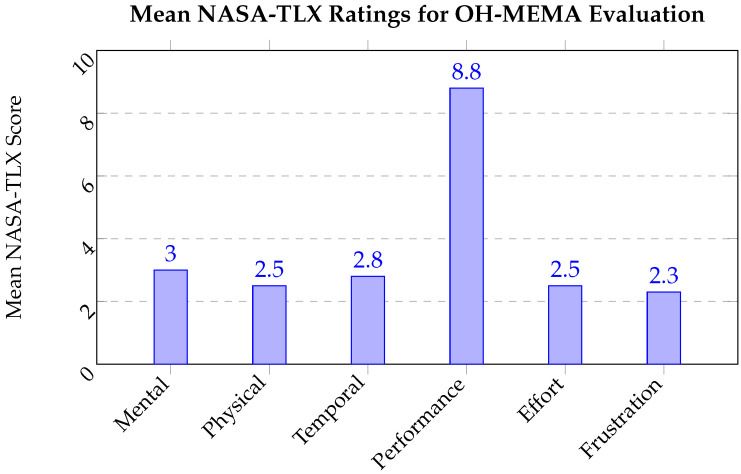
Average perceived workload ratings from four participants using the OH-MEMA tool. Low demand and frustration scores indicate low cognitive load, while high performance scores reflect strong task success.

Four participants representing public health and disease surveillance domains completed the evaluation: two epidemiologists and two disease surveillance analysts. The NASA Task Load Index (NASA-TLX) scores across six workload dimensions are summarized in Figure 6.

Overall, participants reported low cognitive and physical workload while using the OH-MEMA tool. Average mental demand (3.0), physical demand (2.5), and temporal demand (2.8) were all low, indicating that the interface did not require excessive effort or time pressure. The performance dimension averaged 8.8, suggesting that users felt highly successful in completing the assigned modeling tasks. Similarly, low average effort (2.5) and frustration (2.3) scores suggest that users found the interface intuitive and efficient.

As shown in Figure 6, the visual summary of NASA-TLX results highlights a clear contrast between the high perceived performance and low workload dimensions. This pattern reinforces that participants were able to perform complex modeling operations with minimal cognitive strain. The only participant reporting higher workload scores (7 across mental, physical, and temporal demand) noted difficulty interpreting predictor variables, suggesting that the higher effort was related to data interpretation rather than the usability of the interface itself.

### 6.7. Qualitative Feedback

Participants described OH-MEMA as effective and easy to use. They valued features such as interactive dual-axis charts, hover tooltips, and the ability to switch between univariate and multivariate models. Users recommended clearer predictor descriptions and inclusion of additional factors (e.g., confidence intervals).

All participants indicated that OH-MEMA would make sense within their analytical workflow, particularly for data exploration, model interpretation, and hypothesis generation in surveillance contexts. One epidemiologist noted that the tool supports both “the exploration phase of understanding the data” and “assessing which One Health factors have a stronger impact on outcomes of interest.”

Overall, the qualitative feedback reflects aggregated observations from all participants and complements the quantitative usability assessment. Users achieved high task performance with a low workload, confirming the usability and analytical value of OH-MEMA. Feedback-informed interface refinements to improve labeling, documentation, and advanced analytical options, strengthening its potential as a visual analytics system for One Health surveillance.

## 7. Discussion

Our study highlights the advantages of integrating multiple One Health data sources, human clinical data, wastewater pathogen concentrations, and weather variables to improve public health syndromic surveillance and epidemic/pandemic detection, prediction, and management at the county level. The combination of these heterogeneous datasets, facilitated by our visual analytics system, enables a more nuanced understanding of the complex drivers behind disease dynamics, thereby improving predictive accuracy.

The significant improvements in prediction performance achieved by applying STL decomposition indicate that separating the seasonal and trend components from the raw time series data helps reveal underlying patterns and reduces noise, thereby enhancing model robustness. To ensure these gains were not due to information leakage or overfitting, STL decomposition was applied exclusively within the training portion of each rolling time-series split, and model performance was evaluated on held-out validation data. Across multiple cross-validation folds, STL-based models consistently showed lower MAE and RMSE and higher Spearman correlation, suggesting that the decomposition captures meaningful temporal structure rather than artificially smoothing the data [38].

Moreover, our exploration of different random effect groupings revealed that incorporating multiple hierarchical factors, specifically county, month, and age group, was associated with improved predictive performance compared to simpler random structures. This suggests that spatial, temporal, and demographic heterogeneities contribute to variation in COVID-19 hospitalization rates. Including these random effects not only improves model fit but also provides a more realistic representation of the underlying data-generating process.

Our visual analytics system is essential for enabling domain experts to explore and compare multiple model configurations interactively. By providing transparent access to model parameters, random effect choices, and performance metrics, it promotes explainability and facilitates informed decision-making. This transparency is critical for public health applications, where trust and understanding of predictive models are essential. Also, integrating provenance visualization into our visual analytics framework not only enhances transparency and reproducibility but also empowers domain experts to make data-driven decisions with confidence, which is essential in the high-stakes context of pandemic response and One Health surveillance.

However, several limitations remain. The temporal resolution and data completeness vary across datasets, which may impact model stability. In addition, while our system performs well with the current scope of county-level data across several variables, its scalability under larger or real-time data scenarios, such as national-level syndromic surveillance, has not yet been fully evaluated.

To address this, the system has been designed using modular data processing and model execution pipelines that can be adapted to parallel and distributed computing environments. Preliminary tests suggest that pre-processing tasks, such as STL decomposition and mixed-effects modeling, can be batch-executed across counties or demographic groups, allowing for future scalability. However, future work will focus on formal benchmarking, database optimization, and integration of real-time data ingestion to support public health operations at scale.

Future work could also explore the integration of more granular spatial and demographic data, including mobility patterns and vaccination rates, to further enhance predictive capabilities.

Furthermore, benchmarking OH-MEMA against established approaches, including generalized additive mixed models, distributed lag nonlinear models, and hierarchical Bayesian frameworks, is identified as a key direction for future work. Formal comparisons will assess predictive performance, interpretability, and robustness across different diseases and datasets, ensuring that the framework provides competitive advantages in real-world surveillance scenarios.

While our case studies focus on COVID-19 in Oklahoma, the OH-MEMA framework is largely disease-agnostic. Core modules such as data preprocessing, visualization, mixed-effects modeling, and interactive model comparison are applicable to other infectious diseases, including influenza, RSV, and other syndromic surveillance targets, provided similar temporal and predictor datasets are available. Some components, such as feature selection, time-series decomposition, and random-effects specification, are inherently general and do not depend on a specific disease. We note that data sparsity or extensive missing values may affect model stability and visualization clarity; however, the system’s preprocessing options, imputation strategies, and human-in-the-loop workflow provide flexibility to accommodate diverse datasets while maintaining analytical utility across different diseases and regions.

Overall, our approach demonstrates the promise of combining One Health data streams with advanced statistical modeling and interactive visualization to support timely and actionable public health surveillance and intervention. By offering an explainable and scalable decision-support platform, this work contributes toward building more resilient and responsive systems for future epidemic and pandemic response.

## 8. Conclusions

In this paper, we presented OH-MEMA, an interactive visual analytics system that enables transparent and interactive modeling of pandemic-related outcomes using heterogeneous One Health data. Our framework integrates mixed-effects models with seasonal-trend decomposition and visual tools for predictor selection, model configuration, and interpretability. By treating diverse factors, such as wastewater viral concentrations and weather conditions, as fixed effects, and incorporating demographic and geographic groupings as random effects, the system allows users to explore complex, multiscale relationships that impact public health outcomes. To support broader adoption and reproducibility, our framework is publicly available online at https://mema.disc.ourcloud.ou.edu/ (accessed on 1 March 2026).

We demonstrated the utility of our system through two case studies focused on Oklahoma counties between 2020 and 2024. The results show that combining predictors across human and environmental domains significantly improves model performance, especially when seasonal patterns are accounted for using STL decomposition. Our system helps users not only identify relevant predictors but also understand how different random effects influence model performance and interpretability.

In addition to quantitative validation, a qualitative user study involving epidemiologists and disease surveillance analysts confirmed that OH-MEMA is both usable and cognitively lightweight. Participants reported low workload scores (NASA-TLX) and provided positive feedback on the system’s interpretability, interactivity, and workflow integration, reinforcing its suitability for expert use in public health contexts.

The combination of statistical rigor and visual interactivity fosters a white-box modeling experience that supports hypothesis generation, policy analysis, and early warning applications for public health officials and researchers. This work highlights the potential of integrated, explainable modeling platforms to support data-driven decisions in complex and evolving health scenarios.

## Figures and Tables

**Table 1:** Summary of the Oklahoma One Health case-study dataset used in OH-MEMA.

Characteristic	Summary
Geographic scope	Oklahoma, USA (county-level)
Number of counties	77
Weeks/time span	Weekly data from 2020 to 2024
Observation unit after alignment	County-week observations across human clinical, wastewater, and environmental datasets
Human clinical data source	U.S. Centers for Disease Control and Prevention (CDC) and Oklahoma State Department of Health (OSDH)
Wastewater data source	Municipal wastewater pathogen samples collected across Oklahoma counties
Environmental data source	County-aggregated gridMET meteorological data, including weekly mean NET
Outcomes available in the integrated clinical dataset	COVID-19 confirmed cases, hospitalizations, and deaths
Outcomes analyzed in the reported case studies	Case Study I: weekly COVID-19 hospitalizations in Tulsa County; Case Study II: COVID-19 cases
Missing-data handling	Short gaps (≤2 consecutive weeks) were filled by linear interpolation; longer gaps were imputed using county-specific seasonal means; sensitivity analyses compared this strategy with interpolation-only and complete-case analysis
Wastewater variables described	Pathogen quantification including SARS-CoV-2, Influenza, and RSV

**Table 2:** Performance of prediction models with and without STL decomposition. C: County, M: Month, S: Sex group, A: Age group.

Model	Random Effects	MAE	RMSE	Corr.	MAE (STL)	RMSE (STL)	Corr. (STL)
M1: Wastewater	County	0.1399	0.1977	63.71	0.0899	0.1377	66.71
	Month	0.1196	0.1729	75.92	0.0696	0.1029	85.12
	Sex Group	0.1297	0.1955	65.79	0.0927	0.1365	67.79
	Age Group	0.1213	0.1860	64.91	0.0913	0.1360	67.91
	C + M + S	0.1102	0.1496	78.21	0.0802	0.1178	85.32
	C + M + A	0.0827	0.1073	82.50	0.0627	0.1003	86.50
M2: NET	County	0.1802	0.2478	52.32	0.1002	0.1478	66.32
	Month	0.1362	0.1907	66.60	0.0762	0.1007	86.20
	Sex Group	0.1802	0.2478	52.32	0.1151	0.1489	62.32
	Age Group	0.1802	0.2478	52.32	0.1102	0.1478	62.62
	C + M + S	0.1370	0.1908	66.46	0.0802	0.1196	83.21
	C + M + A	0.1370	0.1908	66.46	0.0802	0.1178	85.32
M3: Wastewater + NET	County	0.1208	0.1802	74.21	0.0808	0.1202	77.21
	Month	0.0856	0.1089	84.35	0.0696	0.0989	88.35
	Sex Group	0.1159	0.1777	75.21	0.0807	0.1201	77.10
	Age Group	0.1084	0.1597	75.03	0.0804	0.1137	79.03
	C + M + S	0.0835	0.1084	85.69	0.0735	0.1084	86.69
	C + M + A	0.0673	0.0930	89.47	0.0673	0.0930	89.47

**Table 3:** Performance of prediction models for COVID-19 cases prediction with and without STL decomposition.

Model	Random Effects	MAE	RMSE	Corr.	MAE (STL)	RMSE (STL)	Corr. (STL)
M1 = Wastewater	County	0.0543	0.1021	64.08	0.0443	0.0721	75.08
	Month	0.0313	0.0531	84.04	0.0213	0.0431	89.04
M2 = NET	County	0.0718	0.1342	48.52	0.0518	0.1042	66.52
	Month	0.0486	0.0891	78.85	0.0426	0.0791	81.85
M3 = Wastewater + NET	County	0.0489	0.0922	70.06	0.0489	0.0622	81.06
	Month	0.0313	0.0509	84.28	0.0213	0.0309	91.28

## Data Availability

The system is available at https://mema.disc.ourcloud.ou.edu/ (accessed on 1 March 2026) and can be accessed with the sample dataset or user-uploaded datasets. The code used for data processing, feature engineering, and mixed-effects modeling is publicly available at https://github.com/OU-DISC/mixed-model-creator (accessed on 1 March 2026). The datasets analyzed in this study are available from the corresponding author upon reasonable request, subject to data use agreements and privacy considerations. Additionally, the CSV file used for demonstration and validation is included as Supplementary Material (Supplementary Table S1).

## Supplementary Materials

The following supporting information can be downloaded at: https://www.mdpi.com/article/10.3390/jcm15082966/s1, the CSV file used for demonstration and validation (Table S1. Sample dataset for modeling).

## Abbreviations

The following abbreviations are used in this manuscript:

CDC	Centers for Disease Control and Prevention
MAE	Mean Absolute Error
NASA-TLX	NASA Task Load Index
NET	Net Effective Temperature
OH-MEMA	One Health Mixed-Effects Modeling and Analytics
OSDH	Oklahoma State Department of Health
RMSE	Root Mean Square Error
RSV	Respiratory Syncytial Virus
STL	Seasonal-Trend decomposition using LOESS
